# Supplementary material for: Pharmacophore-Oriented Identification of Potential Leads as CCR5 Inhibitors to Block HIV Cellular Entry
Source: Int J Mol Sci. 2022 Dec 17;23(24):16122. doi: 10.3390/ijms232416122 (PMC9784205; doi:10.3390/ijms232416122)
Supplement: Supplementary file 1 [file ijms-23-16122-s001.zip › ijms-2081701-supplementary.pdf]

International Journal of *Molecular Sciences*

MDPI

Article

Pharmacophore-Oriented Identification of Potential Leads as CCR5 Inhibitors to Block HIV Cellular Entry

Pooja Singh[1,†], Vikas Kumar[2,†], Gihwan Lee[3], Tae Sung Jung[4], Min Woo Ha[5], Jong Chan Hong[1,*], Keun Woo Lee[2,*]

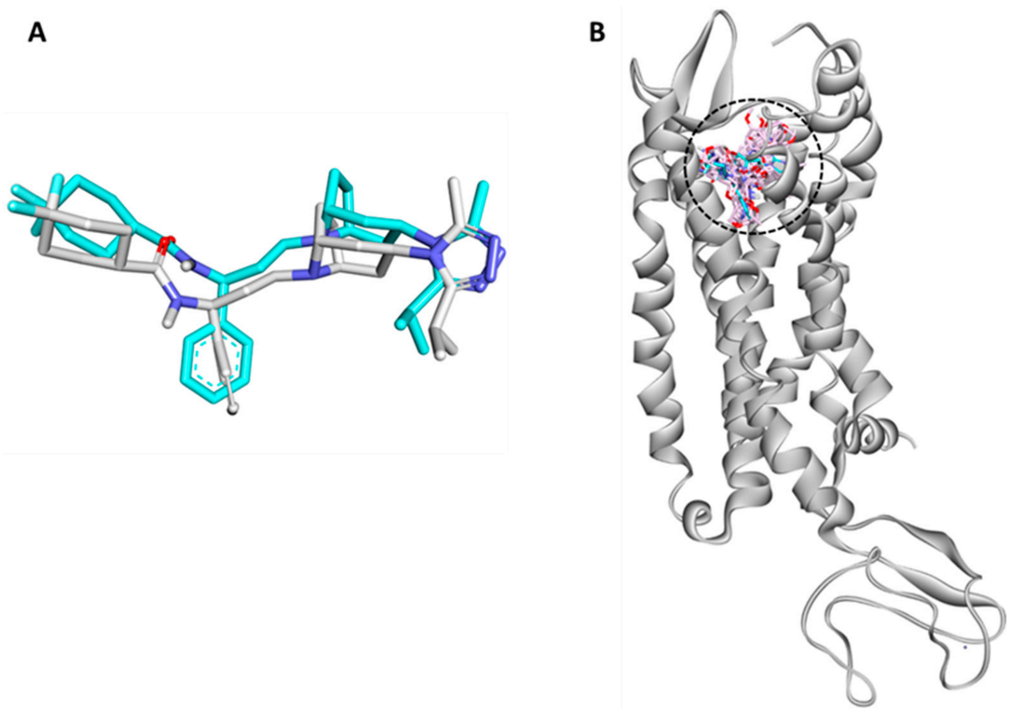

Figure S1. (**A**) Docking parameters validation using co-crystalized structure (grey) and selected docked pose (cyan). The RMSD value between both structures were 1.53Å. (**B**) Binding pattern of MVC (cyan) and 10 potential identified Hits (pink) in the binding cavity of CCR5. Protein is shown in grey color.

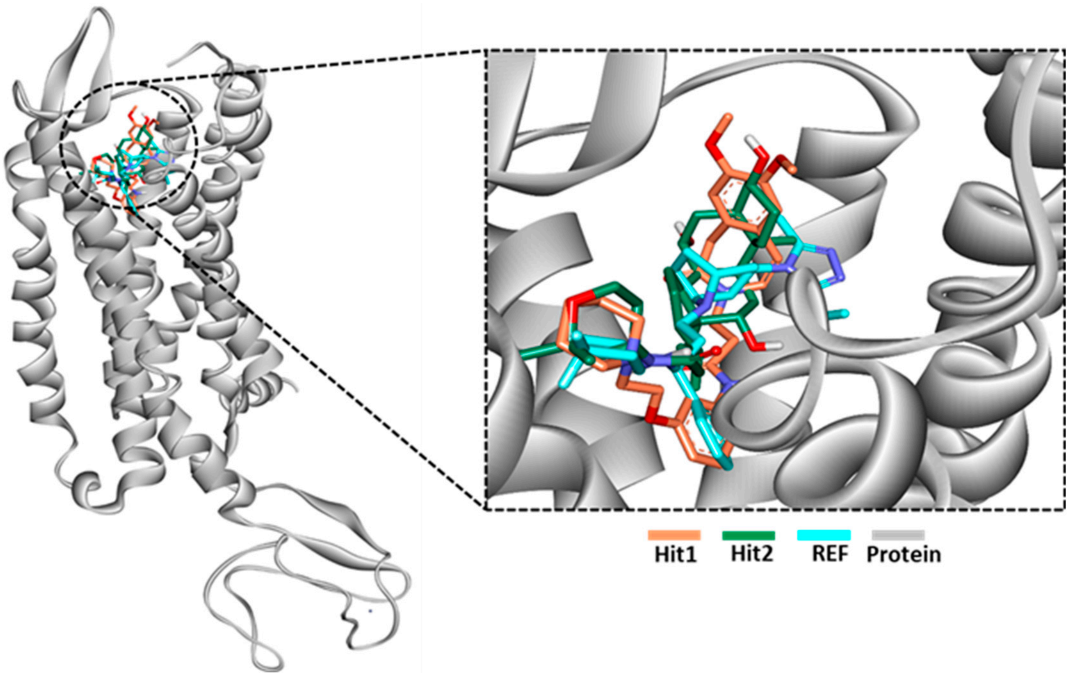

Figure S2. Binding pattern of identified Hits and MVC inhibitor in CCR5 active site. Superimposed view of Hit1, Hit2 and reference is shown in left image and enlarged view is depicted in right side image. The protein is represented in grey whereas Hit. For clear visualization all the hydrogen atoms were deleted except the polar hydrogen atoms.

Table S1. Parameter values used for generation of drug-like database.

**Lipinski's rule of five (ROF)**		**Lipinski's rule of five (ROF)**	
**Parameters**	**Parameters**	**Parameters**	**Cut off**
Number of hydrogen bond donors	Number of hydrogen bond donors	◎ bsorption level	0 (Good)
Number of hydrogen bond acceptors	Number of hydrogen bond acceptors	Solubility level	3 (Good)
Molecular weight (Da)	Molecular weight (Da)	Blood-brain barrier level	3 (Low)
◎ logP value	◎ logP value	CYP2D6 prediction	False
		Hepatotoxic prediction	False

Table S2. List of potential compounds obtained from molecular docking.

Compound	Goldscore	Chemscore	2D structure
Compound 120	72.73	-40.49	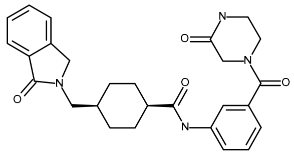
Compound 78112	72.06	-41.30	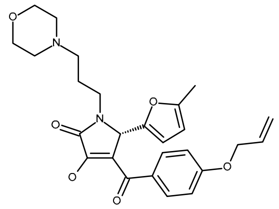
Compound 95427	71.07	-37.47	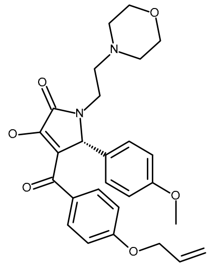
Compound 37501	68.59	-42.58	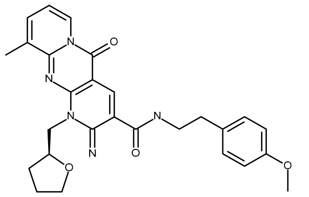
Compound 2074	68.45	-42.57	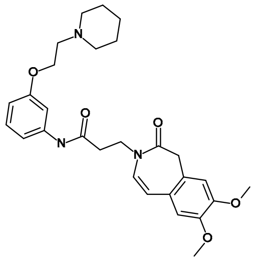

Compound 69088	67.15	-41.87	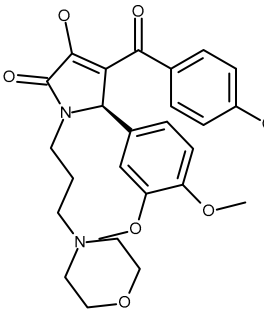
Compound 80978	67.05	-45.07	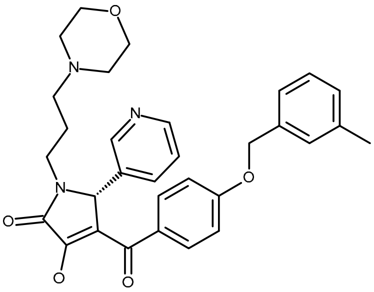
Compound 93533	64.70	-37.92	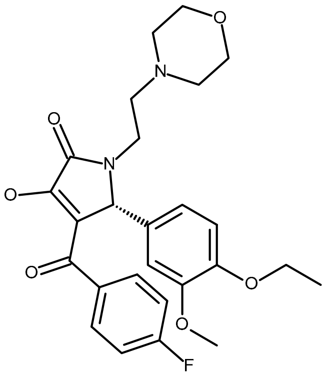
Compound 519	63.26	-41.09	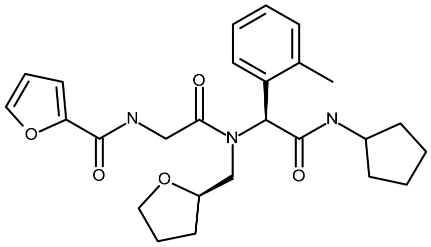

Compound 318	63.01	-37.93	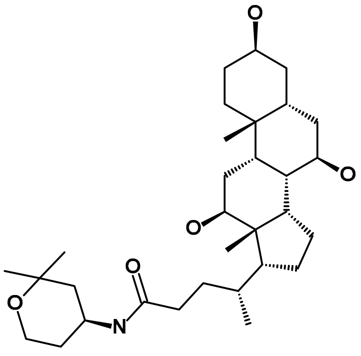
MVC	55.26	-40.47	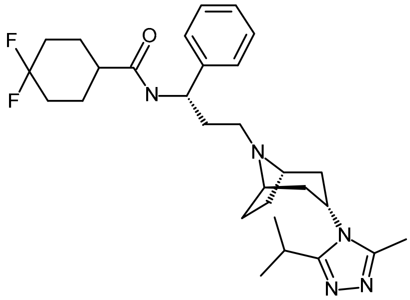

Table S3. Molecular docking and molecular dynamics simulation analysis of final hit compounds of CCR5.

**Systems**	**Docking score**			**Potential Energy**	**Number of Hydrogen bond**	**$\Delta G_{binding}$ kJ/mol)**
	**Goldscore**	**Chemscore**	**Backbone atoms**	**(kJ/mol)**		
MVC	55.26	-40.47	0.31	-1250831	1.02	55.26
Hit1	68.45	-42.57	0.28	-1250461	0.76	68.45
Hit2	63.01	-37.93	0.26	-1250834	1.80	63.01

Table S4. IUP◎ C names and SMILE code of identified Hit1 and Hit2.

Characters	Hit1	Hit2
IUP◎ C name	3-(7,8-dimethoxy-2-oxo-1H-3-benzazepin-3-yl)-N-[3-[2-(1-piperidyl)ethoxy]phenyl]propanamide	(4R)-N-[(4S)-2,2-dimethyltetrahydropyran-4-yl]-4-[(3R,5R,7R,8S,9S,10R,12S,13S,14S,17R)-3,7,12-trihydroxy-10,13-dimethyl-2,3,4,5,6,7,8,9,11,12,14,15,16,17-tetradecahydro-1H-cyclopenta[a]phenanthren-17-yl]pentanamide
SMILE ID	COc1cc2CC(=O)N(CCC(=O)Nc3cccc(OCCN4CCCCC4)c3)C=Cc2cc1OC	C[C@H](CCC(=O)N[C@H]1CCOC(C)(C)C1)[C@H]2CC[C@H]3[C@H]4[C@H](O)C[C@H]5C[C@H](O)CC[C@@]5(C)[C@H]4C[C@H](O)[C@@]23C

Table S5. In silico ◎ DMET properties assessment of MVC and identified CCR5 hit compounds.

ADMET Properties		Hit 1	Hit 2	MVC	Cutoff
◎ bsorption	WS (log mol/L)	-4.53	-5.27	-2.89	< -10 insoluble to < 0 highly soluble
	Caco-2 Permeability (log cm/s)	1.20	0.77	1.45	> 0.90
	I◎ human (% abs)	91.37	85.60	90.16	> 30
	SP (log KP)	-2.87	-2.84	-2.73	> -2.5
	P-glycoprotein Substrate	Yes	Yes	No	Yes/No
	P-glycoprotein I inhibitor	Yes	Yes	No	Yes/No
	P-glycoprotein II inhibitor	No	No	No	Yes/No
Distribution	VDss (human)	0.637	-0.183	0.011	<0.71 low to >2.81 high
	Fraction unbound (human)	0.057	0.054	0.389	Numeric (Fu)
	BBBP (logBB)	-0.844	-0.639	-0.097	> 0.3 high to < -1 poor
	CNS permeability	-2.702	-2.159	-1.377	> -0.2 high to < -3 poor
Metabolism	CYP2D6 substrate	No	No	No	Yes/No
	CYP2D6 inhibitor	Yes	No	No	Yes/No
	CYP3◎ 4 substrate	Yes	Yes	No	Yes/No
	CYP3◎ 4 inhibitor	Yes	No	No	Yes/No
	CYP1◎ 2 inhibitor	No	No	No	Yes/No
	CYP2C19 inhibitor	Yes	No	No	Yes/No
	CYP2C9 inhibitor	No	No	No	Yes/No
Excretion	TC (ml/min/kg)	1.101	0.433	-23.604	Numeric (ml/min/kg)
	Renal OCT2 substrate	Yes	No	No	Yes/No
Toxicity	◎ MES toxicity	No	No	Yes	Yes/No
	Max. tolerated dose (human)	-0.722	-1.956	0.439	>0.477 mg/kg/day
	hERG I inhibitor	No	No	No	Yes/No
	hERG II inhibitor	Yes	No	No	Yes/No
	Oral Rat ◎ cute Toxicity (LD_{50})	2.794	3.746	2.482	Numeric (mol/kg)
	Oral Rat Chronic Toxicity (LO◎ EL)	0.701	-0.136	12.961	Numeric (mg/kg_bw/day)
	Hepatotoxicity	Yes	No	No	Yes/No
	SS	No	No	No	Yes/No
	T. Pyriformis toxicity	0.309	0.301	0.285	>0.5μg/L Toxic
	Minnow toxicity	0.867	1.905	6.291	<-0.3 Toxic
